# Genome-wide identification, characterization, and expression pattern of the late embryogenesis abundant (LEA) gene family in *Juglans regia* and its wild relatives *J. mandshurica*

**DOI:** 10.1186/s12870-023-04096-z

**Published:** 2023-02-06

**Authors:** Jiayu Ma, Dongjun Zuo, Hang Ye, Yujie Yan, Mengdi Li, Peng Zhao

**Affiliations:** 1 Key Laboratory of Resource Biology and Biotechnology in Western China, Ministry of Education, College of Life Sciences, Northwest University, Xi’an, 710069 Shaanxi China; 2 College of Life and Environmental Sciences, University of Birmingham, Edgbaston, Birmingham, B15 2TT UK

**Keywords:** *LEA* gene family, *Juglans*, Whole-genome duplication, Anthracnose resistance

## Abstract

**Background:**

Late Embryogenesis Abundant (LEA) proteins are a class of proteins associated with plant stress resistance. Two *Juglans* species, *Juglans regia* and *J. mandshurica*, are both diploid (2n = 32), monoecious perennial economic tree species with high edible, pharmaceutical, and timber value. The identification, characterization, and expression patterns of *LEA* proteins in *J. regia* and its wild relative, *J. mandshurica*, would not only provide the genetic basis of this gene family, but it would also supply clues for further studies of the evolution and regulating mechanisms of *LEA* proteins in other tree species.

**Results:**

In this study, we identified 25 and 20 members of the *LEA* gene family in *Juglans regia* and its wild relative, *Juglans mandshurica*, respectively. The results of phylogenetic analysis showed that the *LEA* members were divided into eight main subgroups. Predictions of their physicochemical properties showed the variable characteristics of LEA proteins, and the subcellular localization analysis indicated that most LEA proteins are localized in the nucleus. Chromosomal localization analysis and gene replication pattern prediction indicated that WGD is the predominant duplication mode of *LEA* genes. The results of the comparative analysis indicated a high level of collinearity between the two *Juglans* species. Analysis of *cis*-acting elements indicated that *LEA* genes had a relatively wide range of responses to abiotic stresses and phytohormonal processes, particularly in two phytohormones, methyl jasmonate and abscisic acid. Transcriptome profiling and qRT-PCR experiments showed that *JrLEAs* are commonly expressed in leaves, green husks, and male and female flowers, and most *JmLEAs* are more highly expressed in male flowers. We also hypothesized that *JrLEAs* are involved in the process of anthracnose resistance. Anthracnose-resistant varieties of *JrLEAs* presented relatively high expression levels at later stages.

**Conclusion:**

In this study, we provide a theoretical basis for the functional study of *LEA* genes in *J. regia* and *J. mandshurica*. Analysis of *cis*-acting elements and gene expression indicated that *JrLEAs* and *JmLEAs* play important roles in resistance to biotic stresses in these species.

**Supplementary Information:**

The online version contains supplementary material available at 10.1186/s12870-023-04096-z.

## Background

Plants have unique molecular response mechanisms and physiological reactions that mitigate the effects of external stresses upon themselves when negatively affected by the external environment, a process known as plant resilience [1]. External stresses usually include abiotic and biotic stresses, such as drought [2], temperature extremes [3, 4], land salinization [5], UV irradiation [6], pathogenic bacteria, and harmful insects [7]. Water stress is one of the most common environmental stresses that plants suffer.

As osmoprotective and desiccation damage repair agents, Late Embryogenesis Abundant (LEA) proteins, existing widely in the plant kingdom, are a class of dehydration-resistance-associated glycine-rich proteins with low molecular weights (10–30 kDa) [8]. LEA proteins accumulate in seeds with large quantities at later stages of plant embryogenesis development, responding to a variety of abiotic stresses and protecting higher plants from extreme environmental stresses, especially playing a non-negligible role in resisting drought stress. Although the debate is still ongoing regarding the classification of LEA proteins among various species [9], they can be generally classified into the following eight groups according to their conserved structural domains, as follows: LEA_1, LEA_2, LEA_3, LEA_4, LEA_5, LEA_6, Dehydrin, and Seed Maturation Protein (SMP) [10]. The study of the regulatory mechanisms of *LEA* gene expression is of great importance for modern plant molecular biology. LEA proteins are highly tolerant to dry environments in cotton [11]. Subsequently, more and more studies [8] had shown that LEAs play a key role in the tolerance response to drought stress. The overexpression of *OsLEA* can increase the sensitivity and osmotic tolerance of rice to abscisic acid under drought stress [12]. Similarly, the overexpression of *CaLEA1* enhances stomatal sealing and the expression of the related downstream genes in response to drought and salt stress [13]. Furthermore, *AtLEA14* [14] and *SiLEA14* [15] genes have been shown to be salt-tolerant. Recently, *TaLEA* [16], *ZmLEA3* [17], and *SmLEA* [18] have also been shown to be resistant to drought stress.

The Persian (English) walnut (*Juglans regia* L., 2n = 32), also known as the common walnut, belonging to the genus *Juglans* in the family Juglandaceae and the monoecious perennial tree [19], is the second most valuable nut crop in the world, being surpassed only by almonds (*Prunus dulcis*) [20]. In China, *J. regia* is mainly grown and cultivated in the provinces of Xinjiang, Shaanxi, Yunnan, and Hebei [21]. Walnuts have important edible and medicinal value, owing to their dense canopies and leafy branches, their kernel fat content of 65–83%, their protein content of more than 15%, and a variety of essential trace elements. *Juglans mandshurica* (2n = 32), a closely related wild species of the Persian walnut, is an ecologically important, wind-pollinated, and endemic species that grows in northern and northeastern China, Korea, Japan, and the far eastern section of Russia [22–25]. The *LEA* gene family is important for plant growth and developmental processes, and so far, *LEA* genes have been identified in many plants, such as Arabidopsis [14], poplar [26], cucumber [27], and oilseed rape [28]. However, the identification, distribution, and characterization of *LEA* gene family members in *Juglans*, as well as their expression patterns, are still unknown. Hence, *Juglans*, identifying and characterizing *LEA* gene family members across the genomes of the two *Juglans* species and conducting a comprehensive comparative study are necessary.

In the present study, we identified and analyzed the *LEA* gene family for the first time in *J. regia* and its wild relative, *J. mandshurica*. We determined their phylogenetic relationships, predicted their physicochemical properties, identified their conserved structural domains, investigated their chromosomal localization and gene duplication patterns, and performed gene structure analysis and *cis-*acting element analysis for *LEA* gene members in the *J. regia* and *J. mandshurica* genomes. Additionally, we produced *LEA* gene expression profiles using transcriptome sequencing and qRT-PCR based on multi-tissue organs and biotic stress treatment conditions. The results not only provide a fundamental basis for studying *LEA* gene function in *J. regia* and *J. mandshurica*, but they also provide clues for further studies of the evolution and regulating mechanisms of *LEA* proteins in other tree species.

## Materials and methods

### Identification of *LEA* gene members

To identify the candidate members of the *LEA* gene family in two *Juglans* species, 51 LEA protein sequences of *Arabidopsis thaliana* (https://www.arabidopsis.org/) were adopted as query sequences to perform the genome-wide BLASTP in the *J. regia* and *J. mandshurica* [29]. Subsequently, the protein structural domains were analyzed using the CDD (https://www.ncbi.nlm.nih.gov/Structure/bwrpsb/bwrpsb.cgi), Pfam (http://pfam.xfam.org/), and SMART (http:/ /smart.embl.de/ /smart.embl.de/) databases, respectively. The candidate genes which contained any of the LEA_1, LEA_2, LEA_3, LEA_4, LEA_5, LEA_6, SMP, or Dehydrin structural domains were considered to be members of *LEA* gene family. Afterward, the identified *JrLEAs* and *JmLEAs* were submitted to the online website of GSDs (http://gsds.gao-lab.org/) for gene structure analysis and visualized using TBTOOLS software [30].

### Prediction of physicochemical properties and subcellular localization

Physicochemical properties of all identified *LEA* proteins in *J. regia* and *J. mandshurica* were predicted using the ExPASy online tools (http://www.expasy.org/tools/protparam.html). Subcellular localization of all identified *LEA* genes in *J. regia* and *J. mandshurica* were predicted using the WoLF PSORT online tools (https://wolfpsort.hgc.jp/).

### Characteristics and comparative analysis of the identified *LEA* genes

Chromosomal localization of all identified *LEA* genes in *J. regia* and *J. mandshurica* were performed using TBTOOLS software according to the gene annotation [30]. For subsequent investigation, candidate genes were renamed corresponding to their position on the chromosomes. Gene duplication events and collinearity analysis were predicted for *JrLEAs* and *JmLEAs* using MCScanX software [31]. Moreover, the KAKS_CALCULATOR 2.0 software [32] was utilized to calculate Ka/Ks values to judge the selective pressure among the identified gene pairs. Furthermore, *cis*-acting element prediction was executed by using 2000 bp sequences upstream of the identified *LEA* genes via PlantCARE (https://bioinformatics.psb.ugent.be/webtools/plantcare/html/).

### Phylogenetic analysis

The maximum likelihood phylogenetic tree of the *LEA* members in *Arabidopsis thaliana*, *J. regia*, and *J. mandshurica* was constructed by using the IQTREE software [33], in which the VT + F + G4 was adopted as the best-fit substitution model according to the BIC score with 1000 times ultra-fast bootstraps. The phylogenetic trees were visualized using the iTOL online website (https://itol.embl.de/login.cgi).

### Protein-protein interactions and microRNA targeting analysis

Uploading LEA protein sequences of two *Juglans* species to the STRING online website (https://cn.string-db.org) to predict protein-protein interactions. All genomic sequences of identified LEA members were submitted as candidates to predict of potential miRNAs, using the default parameters of psRNATarget online website (https://www.zhaolab.org/psRNATarget/) [34]. Visualization using Cytoscape software default parameters [35].

### Transcriptome profile analysis and qRT-PCR verification

For the transcriptome analysis, multi-tissue gene expression data were obtained from our previously determined transcriptome data [36, 37]. Meanwhile, the walnut disease resistance gene expression data were obtained from the public Sequence Read Archive database (https://www.ncbi.nlm.nih.gov/geo/query/acc.cgi?acc=GSE147083) [38], in which the F26 indicated anthracnose-resistant varieties, while F423 indicated anthracnose-susceptible varieties. Firstly, the raw data were filtered by using the FASTAP software [39], after which the clean reads were mapped to the reference genome of *J. regia* using HISAT2 software [40]. Finally, the expression of genes was calculated using FEATURECOUNTS [41].

Subsequently, the qRT-PCR were implemented to furtherly explore the expression pattern of the identified LEA genes. The leaves, green husks, male and female flowers of the two species in the same developmental period (maturity) were selected for the qRT-PCR verification. A total of 3 biological replicates were present per species/tissue organ. This study was approved by the Chinese government and carried out according to the laws of the People’s Republic of China. All participants had a license approval letter from the College of Life Sciences, Northwest University. Both male and female flowers were harvested in mid-April from the Zhuque National Forest Park (ZNFP) in Xi’an, Shaanxi Province (108°E, 33°N, Altitude 1340.8 m), while the leaves and green husks were harvested from ZNFP in late August. All samples were immediately frozen in liquid nitrogen after collection for further utilization. In this study, Prof. Peng Zhao identified individual *J. mandshurica* and *J. regia* trees according to the following botanical characteristics: leaves, buds, male flowers, female flowers, stems, and fruits. We obtained the permissions to collect those plant samples from ZNFP. The voucher specimen of *J. regia* and *J. mandshurica* (deposition accession numbers: NWU2020016 and NWU2020036) were stored at the Evolutionary Botany Laboratory, College of Life Sciences, Northwest University (Xi’an, Shaanxi, China).

Total RNA was extracted from each sample using an RNA extraction kit (plant RNA Kit (50) OMEGA, USA). Complementary DNA (cDNA) was synthesized using 5× PrimeScript RT Master Mix (Takara) reverse transcriptase. The above cDNA was diluted 5-fold as qRT-PCR template. Subsequently, qRT-PCR experiments were performed on a Bio-Rad CFX96 fluorescent quantitative PCR instrument using 2× Plus SYBR real-time PCR mixture (Biotec) as a fluorescent dye. Each sample had 3 biological replicates and 3 technical replicates. *J. regia* β-Actin was selected as the internal reference gene and primers were designed using the Primer3Plus online website (https://www.primer3plus.com). The primer design sequences are shown in Table S1. qRT-PCR results were analyzed by the 2^-ΔΔCT^ method [42].

## Results

### Genome-wide identification and phylogenetic analysis of *LEA* genes in *Juglans*

A total of 25 *LEA* gene family members in *J. regia* and 20 *LEA* gene family members in *J. mandshurica* were present in the samples, and we renamed all members identified in two *Juglans* species according to their positions on the chromosomes. Details regarding the names, gene IDs, and protein sequences of all identified *J. regia* and *J. mandshurica LEA* members were shown in Table S2.

**Fig. 1:**
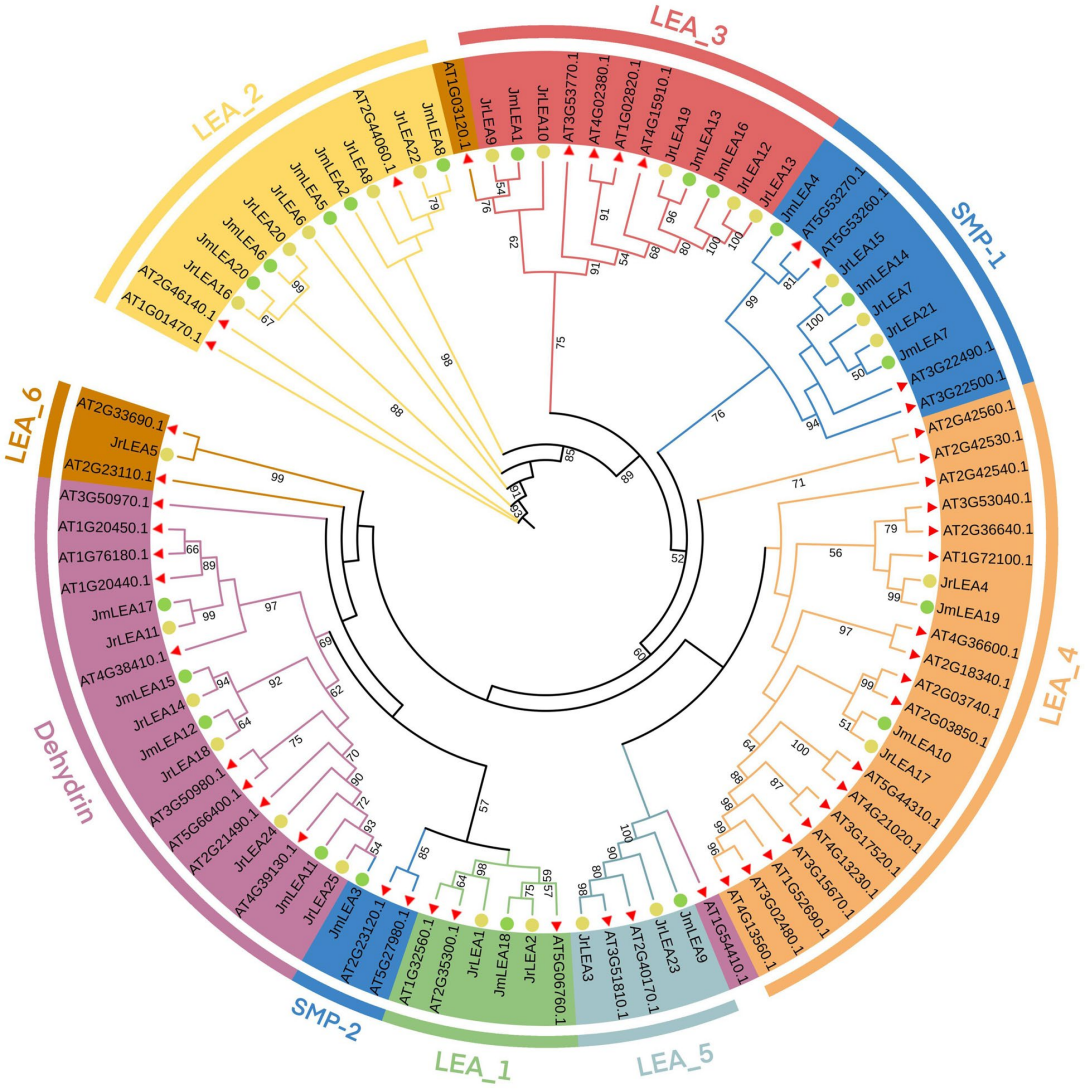
The Maximum Likelihood phylogenetic tree (ML) of LEA protein of *Arabidopsis thaliana*, *Juglans regia* and *Juglans mandshurica*. The red triangles, yellow circles, and green circles represent Arabidopsis, *J. regia*, and *J. mandshurica*, respectively

The maximum likelihood (ML) tree was reconstructed for the *LEA* members in *J. regia* and *J. mandshurica*. All members of the *LEA* family of *Arabidopsis*, *J. regia* and *J. mandshurica* could be divided into eight groups with high bootstraps value (=1000), as follows: LEA_1, LEA_2, LEA_3, LEA_4, LEA_5, LEA_6, SMP, and Dehydrin, each of which corresponded to a unique domain (Fig. 1). Therein, the largest subgroup was LEA_4, which contained 2 *JrLEA*s, 2 *JmLEA*s, and 18 *AtLEA*s, followed by the Dehydein subgroup, which contained 5 *JrLEA*s, 4 *JmLEA*s, and 19 *AtLEAs*. Both the LEA_2 and SMP groups had 13 *LEA* members, with the former containing 5 *JrLEA*s, 5 *JmLEA*s and 3 *AtLEA*s, and the latter included 3 *JrLEA*s, 4 *JmLEA*s, and 6 *AtLEA*s, respectively. The rest members of *LEA* were divided into other groups, in which 5 *JrLEA*s, 3 *JmLEA*s, and 4 *AtLEA*s were distributed in group LEA_3, 2 *JrLEA*s, 1 *JmLEA,* and 3 *AtLEA*s were distributed in group LEA_1, 2 *JrLEA*s, 1 *JmLEA,* and 2 *AtLEA*s were distributed in group LEA_5, and 1 *JrLEA* and 3 *AtLEA*s were distributed in group LEA_6. No mixture was present among each identified group, indicating that the LEA gene family members were relatively conserved within different groups of these three species.

### Physicochemical properties and subcellular localization

The length of the LEA proteins in *J. regia* ranged from 88 amino acids (JrLEA5) to 534 amino acids (JrLEA4), with an average length of 186.28 amino acids (Table 1). LEA proteins in *J. mandshurica* were longer, ranging from 92 amino acids (JmLEA3) to 773 amino acids (JmLEA15), with an average length of 223.45 amino acids. The molecular weights of LEA proteins in *J. regia* ranged from 9659.57 Da (JrLEA5) to 59,259.16 Da (JrLEA4), with an average of 20,219.93 Da. Similarly, the molecular weights of JmLEAs were greater than those of *J. regia*, which ranged from 9815.01 Da (JmLEA3) to 59,504.39 Da (JmLEA19), with an average of 24,325.8545 Da. In addition, 17 and 13 LEA proteins in *J. regia* (JrLEA2, JrLEA3, JrLEA4, JrLEA5, JrLEA6, JrLEA7, JrLEA8, JrLEA11, JrLEA15, JrLEA16, JrLEA18, JrLEA20, JrLEA21, JrLEA22, JrLEA23, JrLEA24, and JrLEA25) and *J. mandshurica* (JmLEA1, JmLEA2, JmLEA3, JmLEA4, JmLEA5, JmLEA6, JmLEA7, JmLEA8, JmLEA11, JmLEA12, JmLEA17, JmLEA18, and JmLEA19) were acidic proteins (Isoelectric point < 7), respectively. A total of 11 and 7 LEA proteins were present in *J. regia* and *J. mandshurica*, respectively, with instability index values > 40. Almost all identified LEA proteins in *J. regia* and *J. mandshurica* had a positive Grand Average of Hydropathicity (GRAVY) with only five exceptions (*JrLEA16, JrLEA20, JmLEA1, JmLEA5*, and *JmLEA6*), which suggested that the majority of LEA proteins in *J. reiga* and *J. mandshurica* were hydrophobic. In addition, the identified *LEA* members were distributed in mitochondria, chloroplasts, the nucleus, the cytoskeleton, peroxisomes, and the extracellular matrix. Most of the identified *LEA* members were located in the nucleus (Table 1).

**Table 1:** Information on physicochemical properties and prediction of subcellular localization of LEA proteins from *J. regia* and *J. mandshurica*

Gene name	No. of amino acids	Mol. Wt (Da)	Isoelectric point (pI)	Instability index (II)	Aliphatic index	Grand average of hydropathicity (GRAVY)	Subcellular localization^a^
JrLEA1	109	11,668.33	9.64	52.74	56.70	−0.794	mito
JrLEA2	133	13,720.07	6.85	30.22	41.43	−0.952	mito
JrLEA3	113	12,300.39	5.58	43.22	45.84	−1.342	mito
JrLEA4	534	59,259.16	5.26	38.02	49.42	−1.348	nucl
JrLEA5	88	9659.57	5.15	60.89	41.14	−1.340	mito
JrLEA6	151	16,362.01	4.46	18.00	119.40	−0.313	cyto
JrLEA7	276	28,650.05	5.12	44.95	79.64	−0.251	chlo
JrLEA8	319	35,430.31	4.71	18.90	92.82	−0.405	cyto
JrLEA9	94	10,608.21	8.05	52.69	80.96	−0.462	mito
JrLEA10	91	10,290.95	9.45	40.73	76.15	−0.463	chlo
JrLEA11	239	27,208.19	5.36	52.56	53.01	−1.495	nucl
JrLEA12	102	10,666.10	9.25	37.61	75.69	−0.175	chlo
JrLEA13	102	10,066.10	9.25	37.61	75.69	−0.175	chlo
JrLEA14	220	24,182.17	8.94	42.09	46.09	−1.305	nucl
JrLEA15	261	27,118.15	5.02	31.73	73.10	−0.392	nucl
JrLEA16	151	16,317.76	5.11	16.36	109.07	0.042	cyto
JrLEA17	242	27,426.54	9.49	25.87	66.57	−0.947	chlo
JrLEA18	180	19,674.30	6.50	31.61	49.33	−1.152	nucl
JrLEA19	98	10,513.91	9.15	35.11	83.67	−0.219	chlo
JrLEA20	151	16,454.95	4.65	14.42	111.72	0.089	cyto
JrLEA21	258	26,423.15	4.77	40.70	79.19	−0.284	chlo
JrLEA22	317	35,092.64	4.70	23.97	95.55	−0.409	cyto
JrLEA23	93	10,118.01	5.92	46.12	47.31	−1.253	nucl
JrLEA24	160	17,381.93	5.93	28.42	45.75	−1.219	nucl
JrLEA25	175	18,905.30	5.58	43.24	44.57	−1.396	nucl
JmLEA1	122	13,527.49	6.42	54.66	86.39	0.315	mito
JmLEA2	203	22,561.99	4.89	11.99	99.36	−0.070	pero
JmLEA3	92	9815.01	5.26	56.20	59.46	−0.577	cyto
JmLEA4	180	18,702.05	5.59	21.90	97.61	−0.007	extr
JmLEA5	152	16,481.26	4.85	17.75	121.84	0.272	cyto
JmLEA6	151	16,483.00	4.65	14.42	112.98	0.105	nucl
JmLEA7	181	18,669.53	4.42	45.50	75.58	−0.281	chlo
JmLEA8	284	31,497.04	4.87	22.45	107.99	−0.174	cyto
JmLEA9	110	12,158.44	7.91	54.56	57.64	0.995	chlo
JmLEA10	121	13,941.73	9.46	38.50	53.31	−1.321	nucl
JmLEA11	175	18,889.21	5.28	46.83	42.91	−1.408	nucl
JmLEA12	180	16,978.33	6.80	32.44	46.61	−1.148	nucl
JmLEA13	98	10,499.88	9.15	36.37	83.67	−0.247	chlo
JmLEA14	474	51,526.75	9.11	43.20	80.80	−0.358	mito
JmLEA15	773	85,676.10	7.31	39.69	46.27	−1.378	nucl
JmLEA16	101	10,702.11	9.45	36.46	75.45	−0.303	chlo
JmLEA17	244	27,510.36	5.46	53.28	49.18	−1.577	nucl
JmLEA18	125	12,918.24	6.32	30.63	48.64	−0.898	mito
JmLEA19	537	59,504.39	5.14	33.78	50.20	−1.313	nucl
JmLEA20	166	18,474.18	7.77	17.67	91.02	−0.175	nucl

*mito* Mitochondrion, *nucl* Nucleus, *cyto* Cytoskeleton, *chlo* Chloroplast, *pero* Peroxisome, *extr* Extracell

### Chromosome location and duplication patterns

**Fig. 2:**
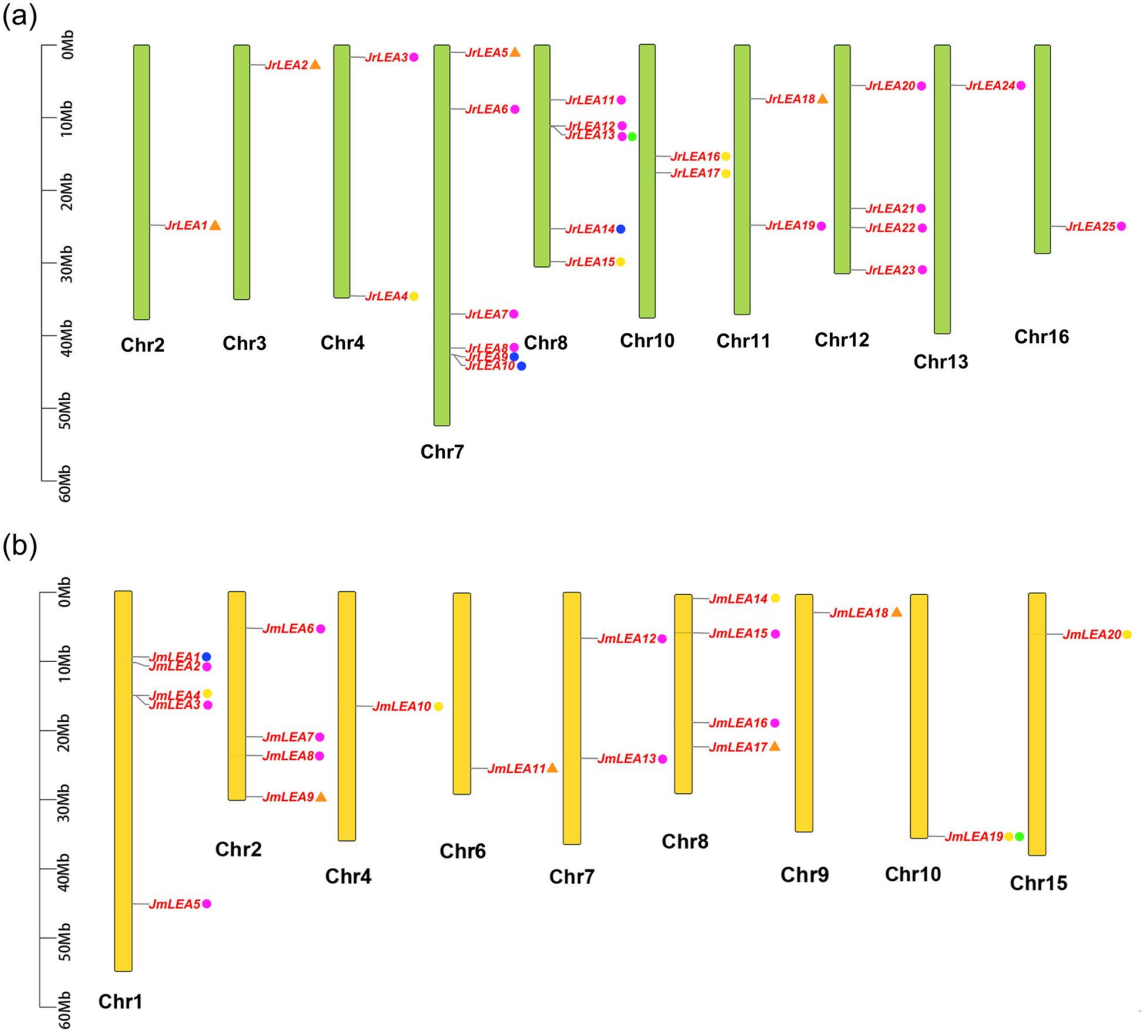
Chromosomal distribution and duplication types of *LEA* genes. **a** Chromosomal localization and duplication types of the *LEA* genes in *J. regia*; **b** Chromosomal localization and duplication types of the *LEA* genes in *J. mandshurica*. The orange triangles, purple circles, blue circles, yellow circles, and green circles represent Singleton, Whole genome duplication (WGD), Tandem duplication (TD), Dispersed duplication (DSD), and Proximal duplication (PD), respectively

For *J. regia*, a total of 25 *LEA* genes were distributed on 10 different chromosomes (Fig. 2a). Chromosome 7 contained the greatest number of *LEA* genes (*JrLEA5*, *JrLEA6*, *JrLEA7*, *JrLEA8*, *JrLEA9*, and *JrLEA10*), followed by chromosome 8 (containing *JrLEA11*, *JrLEA12*, *JrLEA13*, *JrLEA14*, and *JrLEA15*). For *J. mandshurica* (Fig. 2b), chromosome 1 contained the greatest number of *LEA* genes (*JmLEA1*, *JmLEA2*, *JmLEA3*, *JmLEA4*, and *JmLEA5*). Chromosome 2 (*JmLEA6*, *JmLEA7*, *JmLEA8*, and *JmLEA9*) and chromosome 8 (*JmLEA14*, *JmLEA15*, *JmLEA16*, and *JmLEA17*) both possessed four *LEA* genes, and chromosome 7 had two *LEA* genes (*JmLEA12* and *JmLEA13*). The remaining *LEA* genes were located in unique chromosomes.

The results of gene duplication analysis showed four patterns of *LEA* gene duplication, of which Whole Genome Duplication (WGD) was the major mode in *Juglans* (Fig. 2; Table 2). A total of 14 out of 25 *LEA* genes (56%) in *J. regia* and 10 out of 20 *LEA* genes (50%) in *J. mandshurica* experienced WGD. Furthermore, the Tandem Duplication (TD) mode was found in *JrLEA9*, *JrLEA10*, *JrLEA14*, and *JmLEA1*. Four *JrLEAs* (*JrLEA4*, *JrLEA15*, *JrLEA16*, and *JrLEA17*) and five *JmLEAs* (*JmLEA4*, *JmLEA10*, *JmLEA14*, *JmLEA19*, and *JmLEA20*) underwent Dispersed Duplication (DSD). Notably, several *LEA* genes, identified as singleton genes, did not appear to show duplication, and the *JrLEA13* and *JmLEA19* were considered to have Proximal Duplication (PD).

**Table 2:** Replication types of *LEA* genes in *J. regia* and *J. mandshurica*

Gene name	Singleton	Whole genome duplication (WGD)	Tandem duplication (TD)	Dispersed duplication (DSD)	Proximal duplication (PD)
*JrLEA1*	√				
*JrLEA2*	√				
*JrLEA3*		√			
*JrLEA4*				√	
*JrLEA5*	√				
*JrLEA6*		√			
*JrLEA7*		√			
*JrLEA8*		√			
*JrLEA9*			√		
*JrLEA10*			√		
*JrLEA11*		√			
*JrLEA12*		√			
*JrLEA13*		√			√
*JrLEA14*			√		
*JrLEA15*				√	
*JrLEA16*				√	
*JrLEA17*				√	
*JrLEA18*	√				
*JrLEA19*		√			
*JrLEA20*		√			
*JrLEA21*		√			
*JrLEA22*		√			
*JrLEA23*		√			
*JrLEA24*		√			
*JrLEA25*		√			
*JmLEA1*			√		
*JmLEA2*		√			
*JmLEA3*		√			
*JmLEA4*				√	
*JmLEA5*		√			
*JmLEA6*		√			
*JmLEA7*		√			
*JmLEA8*		√			
*JmLEA9*	√				
*JmLEA10*				√	
*JmLEA11*	√				
*JmLEA12*		√			
*JmLEA13*		√			
*JmLEA14*				√	
*JmLEA15*		√			
*JmLEA16*		√			
*JmLEA17*	√				
*JmLEA18*	√				
*JmLEA19*				√	√
*JmLEA20*				√	

### Conserved structural domains and gene structure analysis

**Fig. 3:**
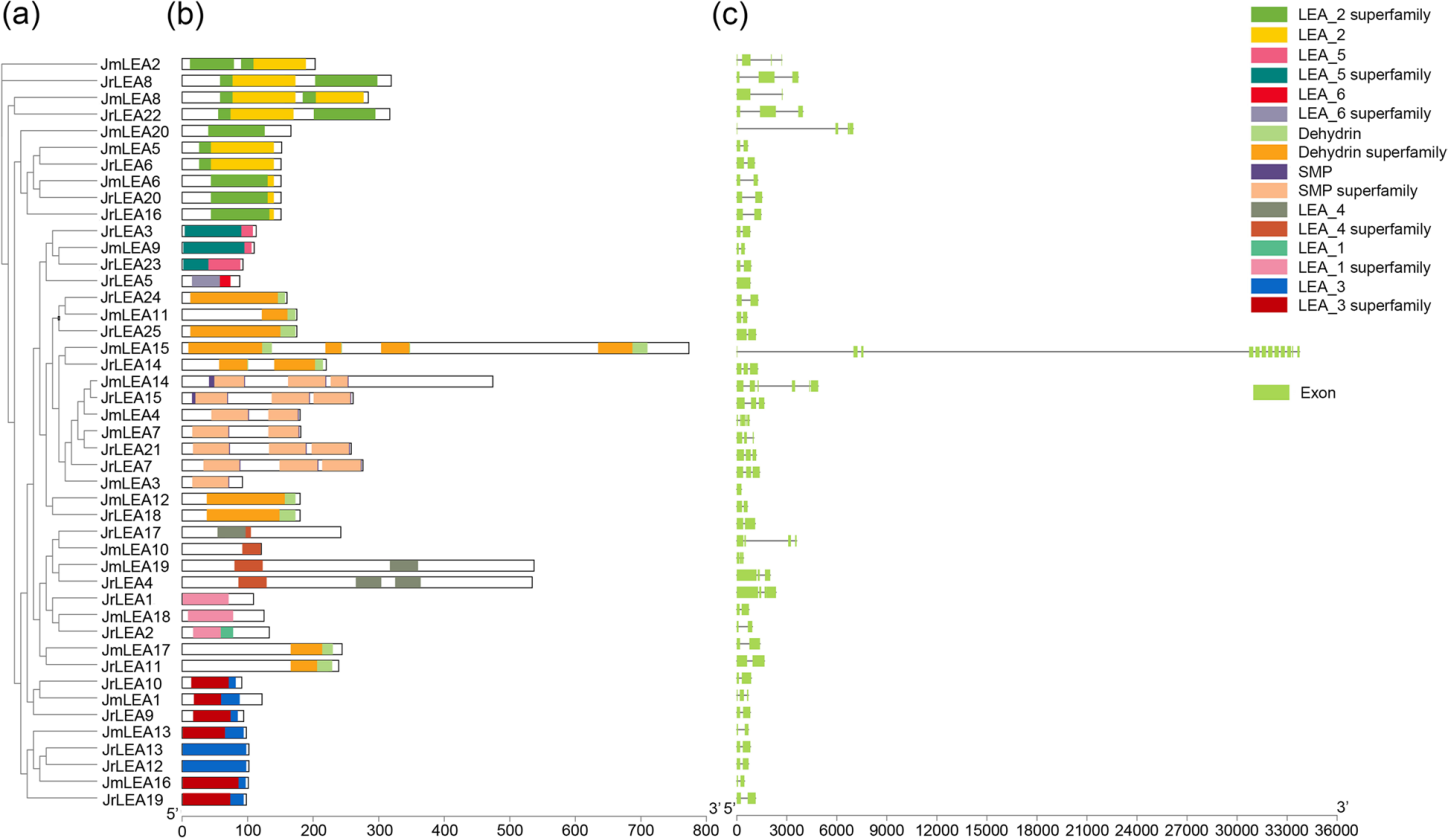
The gene structures and protein domains of LEA members. **a** Maximum likelihood phylogenetic tree of *LEAs* in two *Juglans* species; **b** Protein domains of LEAs in two *Juglans* species. Different domains were represented by different colored boxes; **c** Gene structures of *LEAs* in two *Juglans* species. Green boxes indicated exons, and gray lines indicate introns

To precisely analyze the protein structural domains and gene structures of *J. regia* and *J. mandshurica*, we first constructed a phylogenetic tree of the two species using their LEA protein sequences (Fig. 3a). The reconstructed ML tree based on the two species presented similar topologies compared to those when plusing the *LEA* members of Arabidopsis (Fig. 1). The results show that LEA proteins contained highly conserved LEA protein structural domains and kinase structural domains (Fig. 3b). All members of *LEA* in *J. regia* and its wild relative, *J. mandshurica*, contained an *LEA* signature protein structural domain and a corresponding kinase structural domain. The LEA domain was usually located at the C-terminus of the LEA protein, and the kinase structural domain was usually located at the N-terminal. These results may be related to the functions of LEA proteins. However, the results of gene structure analysis (Fig. 3c) show that the *LEA* gene structures of *J. regia* and *J. mandshurica* were highly divergent. Notably, all *LEA* genes contained multiple exons except for *JrLEA5* and *JmLEA12*, which contained only one exon. *JmLEA15* contained 12 exons; *JmLEA4* and *JmLEA14* contained 6 exons; *JrLEA17*, *JmLEA2*, and *JmLEA20* contained 4 exons; *JrLEA4*, *JrLEA7*, *JrLEA8*, *JrLEA15*, *JrLEA20*, *JrLEA21*, *JrLEA22*, *JmLEA1*, *JmLEA10*, *JmLEA19*, and *JmLEA20* contained 3 exons; and the remaining *LEA* genes contained 2 exons. Some individual *LEA* genes had long introns, especially *JmLEA15*.

### Collinearity and selective pressure analysis

**Fig. 4:**
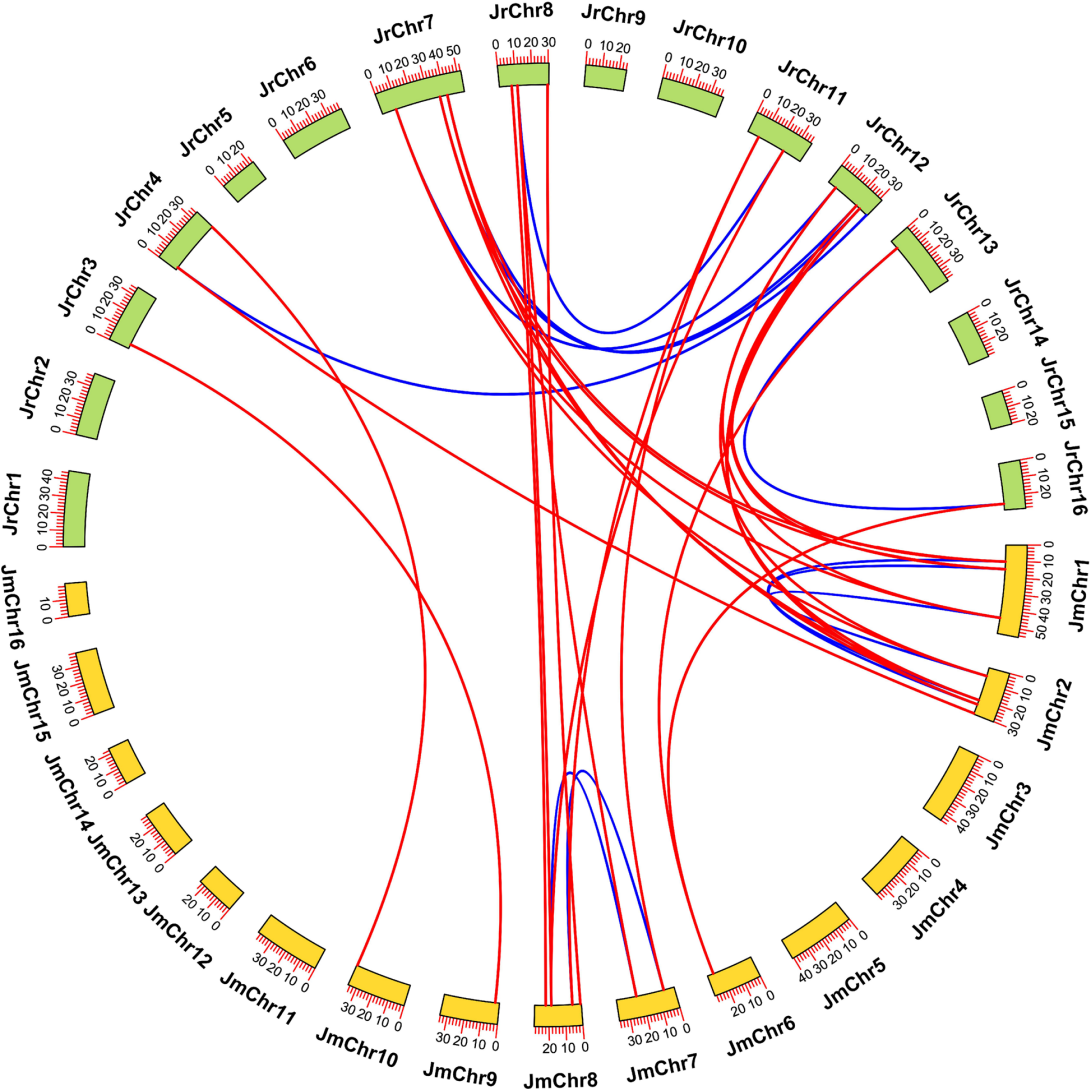
Genome-wide synteny analysis for *LEA* genes among *J. regia* and *J. mandshurica*. Red lines indicate orthologous gene pairs; blue lines indicate paralogous gene pairs

The collinearity predictions showed six and five *LEA* paralogous gene pairs in *J. regia* and *J. mandshurica*, respectively, and 24 *LEA* orthologous gene pairs between the two *Juglans* species (Fig. 4; Table S3). The number of identified orthologous gene pairs was greater than that of paralogous gene pairs, indicating a high degree of collinearity in *LEA* members between two *Juglans* species and indicating that most members of the *LEA* gene family likely existed in the ancestors of the two *Juglans* species rather than being formed separately after their divergence. Between the two *Juglans* species, seven *JrLEAs* (*JrLEA1*, *JrLEA5*, *JrLEA9*, *JrLEA10*, *JrLEA13*, *JrLEA14*, and *JrLEA16*) without collinearity and five *JmLEAs* (*JmLEA4*, *JmLEA10*, *JmLEA11*, *JmLEA17*, and *JmLEA20*) without collinearity were present. Therefore, these genes may be specific to *J. regia* and *J. mandshurica*. In addition, 23 orthologous gene pairs were present between Arabidopsis and *J. regia*, and 15 orthologous gene pairs were present between Arabidopsis and *J. mandshurica* (Table S4). In each of the two *Juglans* species, 10 genes without collinearity were related to *AtLEAs*.

To further explore the selection pressure of the homologous gene pairs of the two *Juglans* species, we subsequently analyzed the Ka/Ks values of these homologous gene pairs (Table S3). The Ka/Ks ratios of most *LEA* homologous gene pairs were less than 1, suggesting that these gene pairs were subject to purifying selection during evolution. The Ka/Ks value of more than 1 for an orthologous gene pair, *JmLEA12* and *JrLEA18*, indicated that it experienced positive selection and might have a relatively fast evolutionary rate.

### Analysis of *Cis*-acting elements in *J. regia* and *J. mandshurica*

**Fig. 5:**
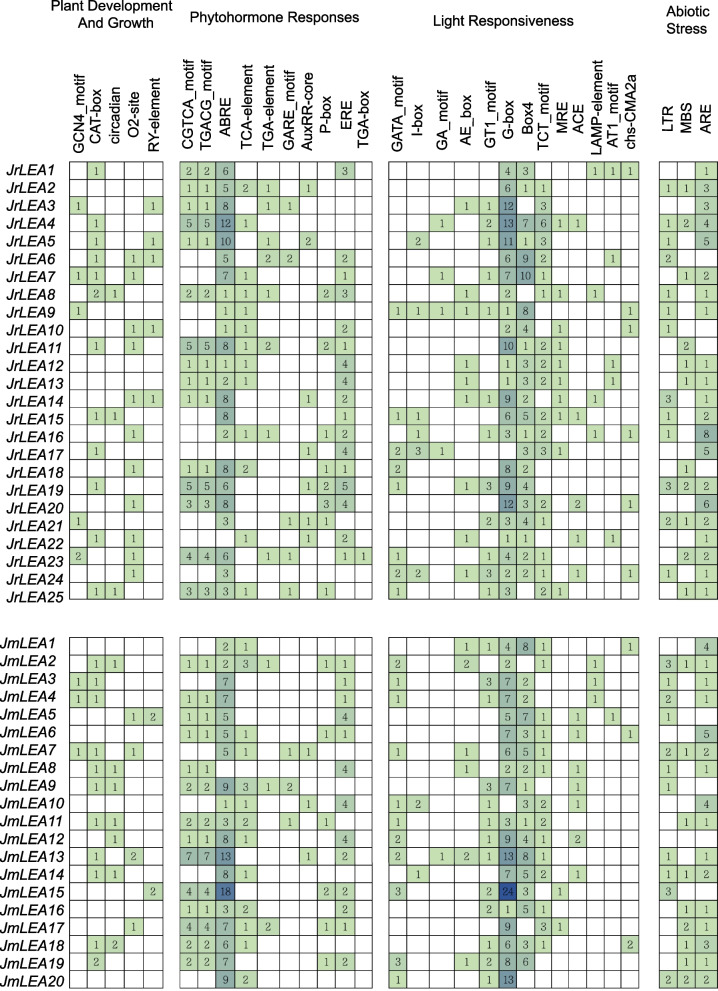
*Cis*-acting elements in promoter regions of *LEA* genes in two *Juglans* species. The number in the colored box represented the number of *cis*-acting elements

To investigate the potential functions of *LEA* genes in *J. regia* and *J. mandshurica*, we analyzed *cis*-acting elements in their upstream promoter regions. We divided the *cis*-acting elements into four main categories, namely those that respond to plant development and growth, plant hormones, abiotic stresses, and light (Fig. 5). The promoter regions of most *LEAs* contained *cis*-acting elements associated with abiotic stress, suggesting that they may play an important role in abiotic stress resistance in the two *Juglans* species. In addition, the promoter regions of *JrLEAs* contained more *cis*-acting elements than those of *JmLEAs*, suggesting that *LEAs* in *J. regia* may be involved in more complex signaling and pathways than those of its wild relative. We also found that *cis*-acting elements in the promoter regions of the *LEAs* of two *Juglans* species contained a large number of elements associated with three phytohormone responses, namely the CGTCA and TGACG motifs (associated with the methyl jasmonate hormone response) and ABRE (associated with the abscisic acid hormone response). *JmLEAs*’ response to light involves more components than that of *JrLEAs*; therefore, *J. mandshurica* is presumably more sensitive to light.

### Protein–protein interactions, microRNA targeting, and expression analysis of *LEA* genes in *J. regia* and its wild relative, *J. mandshurica*

A total of 19 JrLEAs, exclulding JrLEA9, JrLEA10, JrLEA13, JrLEA15, JrLEA17, and JrLEA25, interacted with at least one LEA protein in the two Juglans species. Among them, JrLEA1, JrLEA2, and JrLEA4 had more interactions with other LEA proteins. In contrast, only JmLEA3 interacted with other LEA proteins in *J. mandshurica* (Fig. S1).

**Fig. 6:**
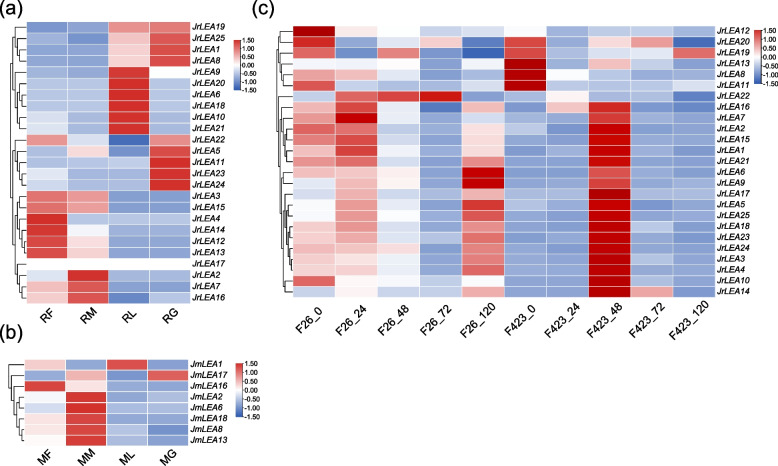
Expression patterns of *LEAs* in *J. regia* and *J. mandshurica*. **a** Expression patterns of identified *LEAs* in female flowers, male flowers, leaves and green husks of *J. regia*; RF, female flowers of *J. regia*; RM, male flowers of *J. regia*; RL, leaves of *J. regia*; RG, green husks of *J. regia*; **b** Expression patterns of identified *LEAs* in female flowers, male flowers, leaves and green husks of *J. mandshurica*; MF, female flowers of *J. mandshurica*; MM, male flowers of *J. mandshurica*; ML, leaves of *J. mandshurica*; MG, green husks of *J. mandshurica*; **c** Expression patterns of identified *LEAs* in *J. regia* under biotic stress. F26 indicated anthracnose-resistant varieties, F423 indicated anthracnose-susceptible varieties. The colored scale reflects gene expression levels

**Fig. 7:**
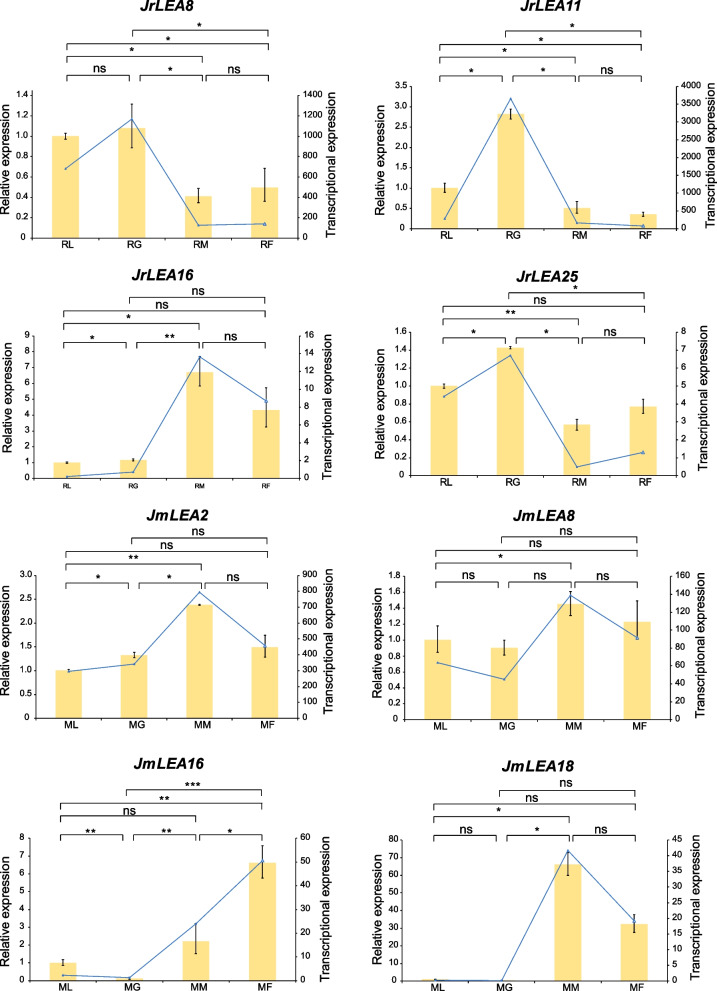
Relative expression of *JrLEAs* and *JmLEAs* in leaves, green husk, male flowers and female flowers. The yellow bars represent data from qRT-PCR experiments, and the blue lines represent transcriptome data. The statistical significance using student’s t-test. ns = no significant different, * = *p* < 0.05, ** = *p* < 0.01, *** = *p* < 0.001

We used transcriptome data from leaves, green husks, and male and female flowers to investigate the expression patterns of *LEA* genes in the two *Juglans* species (Fig. 6a, b, Tables S5 and S6). In addition, we randomly selected four *JrLEA*s and four *JmLEA*s and verified their relative expressions in leaves, green husks, and male and female flowers via qRT-PCR experiments, which were consistent with the transcriptome data (Fig. 7). Validating the results with transcriptome data combined with the qRT-PCR experiments revealed that *JrLEA17* was not expressed in *J. regia*. Ten genes were highly expressed in *J. regia* leaves, eight genes were highly expressed in *J. regia* green husks, nine genes were highly expressed in *J. regia* male flowers, and nine genes were highly expressed in *J. regia* female flowers. However, only eight genes (*JmLEA1*, *JmLEA2*, *JmLEA6*, *JmLEA8*, *JmLEA13*, *JmLEA16*, *JmLEA17*, and *JmLEA18*) were expressed in four selected tissues of *J. mandshurica*. The majority of *JmLEA* genes (7 out of 8) were highly expressed in male flowers. Only one gene, namely *JmLEA1*, was highly expressed in leaves, and *JmLEA17* presented a high expression level in green husks. All of those without collinear *JmLEAs* (unique) were not expressed in female and male flowers, leaves, or green husks. For those without collinear *JrLEAs* (seven in total), JrLEA13 and JrLEA16 were expressed at high levels in male and female flowers, JrLEA9 was expressed at high levels in leaves, and the other four genes (JrLEA1, JrLEA10, JrLEA5, and JrLEA14) were all expressed at low levels in the four selected organs (Fig. S2).

No microRNAs targeted *JrLEA2*, *JrLEA6*, *JrLEA9*, *JrLEA10*, *JrLEA24*, *JmLEA1*, *JmLEA2*, *JmLEA4*, *JmLEA5*, *JmLEA7* or *JmLEA18*. ath-miR414 targeted the most *LEA* genes (including *JrLEA4*, *JrLEA8*, *JrLEA12*, *JrLEA13*, *JrLEA22*, *JmLEA16*, and *JmLEA19*; Fig. S1, Table S7). In addition, ath-miR414 targeted seven *LEA* genes except for *JmLEA19*, which was not expressed in the four selected organs, and *JrLEA8* was highly expressed in green husks. The remaining five genes were all highly expressed in female flowers (Fig. 6).

To further investigate the role of *JrLEAs* in response to biotic stress, we analyzed the gene expression patterns of different varieties of *J. regia* under biotic stress. The results showed that *JrLEAs* were expressed at higher levels in anthracnose-resistant varieties (F26) than in anthracnose-susceptible varieties (F423) after infection (Fig. 6c; Table S8). This indicates that *JrLEAs* might play a role in anthracnose resistance in *J. regia*. Most *JrLEAs* (72%) in anthracnose-susceptible varieties (F423, 48 hours). reached peak expression at 48 h of stress. *JrLEAs* presented a relatively high expression level at the late stage in anthracnose-resistant varieties (F26, 120 hours). Of the seven unique *JrLEAs*, *JrLEA1*, *JrLEA5*, *JrLEA14,* and *JrLEA16* had higher expression levels at 48 hours of infection in variety F423. *JrLEA1* and *JrLEA5* had higher expression levels at 120 hours of infection in variety F26. The other three genes (*JrLEA9*, *JrLEA10*, and *JrLEA13*) showed low expression levels in both F26 and F423 varieties at different periods of infection (Fig. S3). In addition, *JrLEA22* was highly expressed only in anthracnose-resistant varieties, and its expression level increased with time and was significantly different from that of anthracnose-sensitive varieties, indicating that it might be closely associated with the process of anthracnose resistance in walnuts.

## Discussion

The Persian walnut (*J. regia*) and its wild relative, *J. mandshurica*, are both economically valuable tree species [38]. With the development and maturation of genome sequencing technologies in recent years, comparative genomic studies of the two *Juglans* species have become a current research hotspot [11, 29]. Late Embryogenesis Abundant (LEA) proteins are an important class of plant proteins that accumulate mainly at later stages of seed development in response to exogenous stresses [1]. The *LEA* gene family has been extensively studied in plant species, with reports of a total of 51 *LEA* members identified in *Arabidopsis* [14], 84 members identified in the banana [43], and 72 members identified in the sweet orange [44]. We identified a significant difference in the number of the identified *LEAs* in *J. regia* and *J. mandshurica* (Fig. 1). Phylogenetic analysis showed that these *LEA* genes can be divided into eight branches corresponding to their unique structural domains and mRNA homology, indicating that the *LEA* gene family members of these two walnuts are more closely related (Fig. 1). Most LEAs were localized in the cytoplasm and nucleus (Table 1) [45]. The *LEA* gene members of *J. regia* and *J. mandshurica* were distributed heterogeneously across the chromosomes, but some *LEA* gene clusters had high similarity and collinearity (Figs. 2 and 4). This clustering phenomenon might prevent *LEA* genes from losing critical functions as they evolve. The results of the collinearity analysis indicate individual *LEA* genes that were specific to *J. regia* or *J. mandshurica* (Tables S3 and S4). These genes may be related to the specificity of the two *Juglans* species. Most *LEA* homologous gene pairs had Ka/Ks ratios less than 1, suggesting that *JrLEAs* and *JmLEAs* were subject to purifying selection during evolution (Table S3). Duplication events such as segmental type can extend the family members in plant species and modifications as well as point mutations in the gene structure including promoter region and coding sequence site can increase the diversity and modify the expression patterns of new duplicated members [46, 47]. WGD events are a common mechanism for gene duplication [42]. In the present study, we found WGD events to account for the largest proportion of gene duplication categories, corresponding to 56% in *J. regia* and 50% in *J. mandshurica*, suggesting the important role of WGD in the duplication of the *LEA* gene family in the two *Juglans* species (Table 2). However, we observed only a few TD gene pairs (12% in *J. regia* and 5% in *J. mandshurica*), presenting similar proportions observed in other species with TD, accounting for 9.6% in the banana [43] and 6.3% in maize [17]. Although structural domains steadily arose in the identified *LEAs*, they presented variable genic structures in *J. regia* and *J. mandshurica* (Fig. 3).

The *LEA* genes of a variety of plants are involved in response processes to biotic and abiotic stresses [26]. The overexpression of the *OsLEA3–2* gene revealed that, under salt stress or osmotic stress conditions, transgenic rice grew significantly stronger than the control and was able to recover after 20 days of drought stress [12]. The expression profile analysis indicated an important role of the *MwLEA1* gene cloned from *Agropyron mongolicum* in water and salt stresses as well as in abscisic acid regulation [48]. The overexpression of *ZmLEA3* in transgenic tobacco and yeast can enhance tolerance to osmotic and oxidative stress, enabling plants to withstand stress by protecting their protein structures [17]. Despite extensive research in the past, the specific regulation mechanism of LEA proteins that responds to abiotic and biotic stress remains unclear. Investigating the phylogeny, gene structures, and expression profiles of the *LEA* gene family can provide useful information for further studies on *LEA* gene evolution and gene function.

*Cis*-acting elements were involved in the regulation of gene expression, and a large number of *cis*-acting elements detected in this study provided strong evidence for the involvement of *JrLEAs* and *JmLEAs* in abiotic stress responses. We found that the upstream *LEA* promoter region contained several *cis*-acting elements in response to methyl jasmonate (MeJA) and abscisic acid (ABA) (Fig. 5), suggesting that LEA proteins might be involved in plant responses to MeJA and ABA regulation. The synthesis, expression, and physiological activity of LEA proteins are regulated by many factors (e.g., the developmental stage, hormones, ionic changes, and dehydration) and signal transduction pathways [47]. Phytohormones are usually involved in stress responses in plant and are essential for plant adaptation to adverse environmental conditions [49]. Most of the identified plants’ *LEA* gene families have phytohormone-responsive elements, such as tomato [50] and wheat [51]. For example, *LEA* gene expression is regulated by ABA-dependent pathways during seed development and desiccation [8]. Thus, these results suggest that *LEA* genes play an important role in the stress responses of plants.

Perennial crops were once regarded as intractable systems due to their large size and their long juvenile phase and generation length. Recently, the development of perennial crops has been garnering increasing attention, as they are essential components for sustainable agriculture that provide alternative food sources under changing environments. The published high quality *Juglans* genome provided an excellent opportunity to deeply explore the functional network and regulation mechanism of the candidate genes. *LEA* genes were once considered to be responsive to abiotic stress; however, they also play a pivotal role in biotic resistance [17]. Anthracnose causes early tree defoliation at critical times (nut filling and ripening) and leads to alternate-year bearing for nut crops [51], as it is one of the major diseases of walnuts. In our study, in addition to the analysis and discussion of abiotic stress and the analysis of *cis*-acting elements, we also explored LEA expression patterns in various tissues and in anthracnose-resistant varieties (F26), as well as in anthracnose-susceptible varieties (F423).

*JrLEAs* were expressed in leaves, green husks, male flowers, and female flowers, suggesting that these genes play an important role in the development of all four selected tissues. In contrast, most *JmLEAs* might play a role in other organs or in other developmental stages of the selected tissues. *JrLEA8* and *JrLEA19* were highly expressed in both leaves and green husks and had similar expression patterns, suggesting that they might possess the function of synergetic regulation in the development of leaves and green husks. Five *JmLEAs* were highly expressed in the male flowers of *J. mandshurica*, suggesting that these genes are more valuable candidates for studying male flower development in *J. mandshurica* (Fig. 6b; Table S6). The majority of *LEA* genes targeted by ath-miR414 were highly expressed in female flowers, suggesting that it may regulate female flower development by targeting *LEA* genes in the two *Juglans* species (Fig.S1, Table S7). Plants in *Juglans* experience dichogamy [52]. *JrLEA4* and *JrLEA22* (more highly expressed in female flowers than in male flowers) as well as *JrLEA2* and *JrLEA5* (more highly expressed in male flowers than in female flowers) are thought to have a possible direct or indirect effect on dichogamy in *J. regia*. Six genes were more highly expressed in male flowers than in female flowers, and two genes presented the opposite pattern, suggesting that *LEA* genes also influence dichogamy in *J. mandshurica*. The tissue-specific expression of these *LEAs* may be associated with dichogamy in two *Juglans* species, although the regulatory mechanisms involved remain to be further investigated.

In addition, we found higher expression levels of *LEA* genes for anthracnose-resistant varieties (F26) than those for anthracnose-susceptible varieties (F423), indicating that *LEA* genes might play a role in anthracnose resistance in *J. regia* (Fig. 6c; Table S8). Moreover, the expression patterns of genes belonging to the same subgroup were more similar, such as those of *JrLEA15* and *JrLEA21* (SMP), *JrLEA3* and *JrLEA23* (LEA_5), and *JrLEA18* and *JrLEA24* (Dehydrin). The expression levels of most *JrLEA*s increase with time within 48 h after infection [38]. *JrLEA12* and *JrLEA22* were only expressed in F26 varieties, indicating that these two genes are closely associated with anthracnose resistance in *J. regia*. In addition, the *LEA* gene family was also shown to be a gene family associated with disease resistance in *J. mandshurica* [29]. A similar function was found in *ZmLEA3*, which can respond to biotic stresses, and the overexpression of *ZmLEA3* in tobacco results in its increased tolerance to the pathogen pst dc3000 (the pathogen *Pseudomonas syringae* pv. Tomato DC3000) [17]. These results suggest that *LEAs* are not only able to respond to abiotic stresses but also to biotic stresses. *J. regia* is more susceptible to disease than its wild relative, *J. mandshurica* [29]. *J. mandshurica* has been shown to have better resistance to disease to lesion nematode [29]. Therefore, *J. mandshurica* is recommended as a rootstock for *J. regia* to confer disease tolerance/resistance [24, 53–56]. Furthermore, speculation indicates that *JmLEAs* may exhibit better expression for biotic stresses compared with *JrLEAs*, but further studies are still needed in this regard.

## Conclusion

In this study, we systematically identify *LEA* gene family members in *J. regia* and its wild relative, *J. mandshurica.* The phylogenetic analysis showed that the *LEA* genes in *J. regia* and *J. mandshurica* are divided into eight subgroups, similar to results from other plants. The phylogenetic and collinearity analyses indicated that the *LEA* gene family is relatively evolutionarily conserved. The analysis of *cis*-acting elements and gene expression indicated that *JrLEAs* and *JmLEAs* play important roles in resistance to biotic stresses in *J. regia* and *J. mandshurica*. Further exploration is necessary to determine the specific roles of *JrLEAs* and *JmLEAs* in other stresses, such as metal ion stress, extreme temperatures, UV radiation, etc.

## Supplementary Information

**Additional file 1: Figure S1.** Protein interaction network and schematic representation of the regulatory network relationships between the putative miRNAs and their targeted LEA genes. Elliptics represent proteins, rectangles represent miRNAs. The black line indicates protein interactions and the pink line indicates miRNA targeting of LEA genes. **Figure S2.** Expression patterns of *LEA* genes without collinearity in four selected organs of two *Juglans* species. The colored scale reflects gene expression levels. **Figure S3.** Expression patterns of *LEA* genes without collinearity under biotic stress of *J. regia*. F26 indicated anthracnose-resistant varieties, F423 indicated anthracnose-susceptible varieties. The colored scale reflects gene expression levels.

**Additional file 2: Table S1.** Primers for the qRT-PCR experiment. **Table S2.** Protein sequences of all *LEA* genes in *J. regia* and *J. mandshurica*. **Table S3.** Homologous *LEA* gene pairs and Ka/Ks values in *J. regia* and *J. mandshurica*. **Table S4.** Homologous *LEA* gene pairs in Arabidopsis and two *Juglans* species. **Table S5.** The FPKM values of all *LEA* genes in different tissues of *J. regia*. **Table S6.** The FPKM values of all *LEA* genes in different tissues of *J. mandshurica*. **Table S7.** The putative miRNAs and their targeted *LEA* genes. **Table S8.** The FPKM values of all *LEA* genes in anthracnose-resistant varieties (F26) and anthracnose-susceptible varieties (F423) with the time after infection of *J. regia*.

## Data Availability

The raw data were downloaded from the SRA database under accession number (GSE147083).

## Abbreviations

LEA: Late Embryogenesis Abundant
SMP: Seed maturation protein
ML: Maximum likelihood
GRAVY: Grand average of hydropathicity
WGD: Whole genome duplication
TD: Tandem duplication
DSD: Dispersed duplication
