# Supplementary material for: Genome-wide identification, characterization, and expression pattern of the late embryogenesis abundant (LEA) gene family in Juglans regia and its wild relatives J. mandshurica
Source: BMC Plant Biol. 2023 Feb 6;23:80. doi: 10.1186/s12870-023-04096-z (PMC9901102; doi:10.1186/s12870-023-04096-z)

**Additional File 1**

**Figure S1.** Protein interaction network and schematic representation of the regulatory network relationships between the putative miRNAs and their targeted LEA genes. Elliptics represent proteins, rectangles represent miRNAs. The black line indicates protein interactions and the pink line indicates miRNA targeting of LEA genes.

**
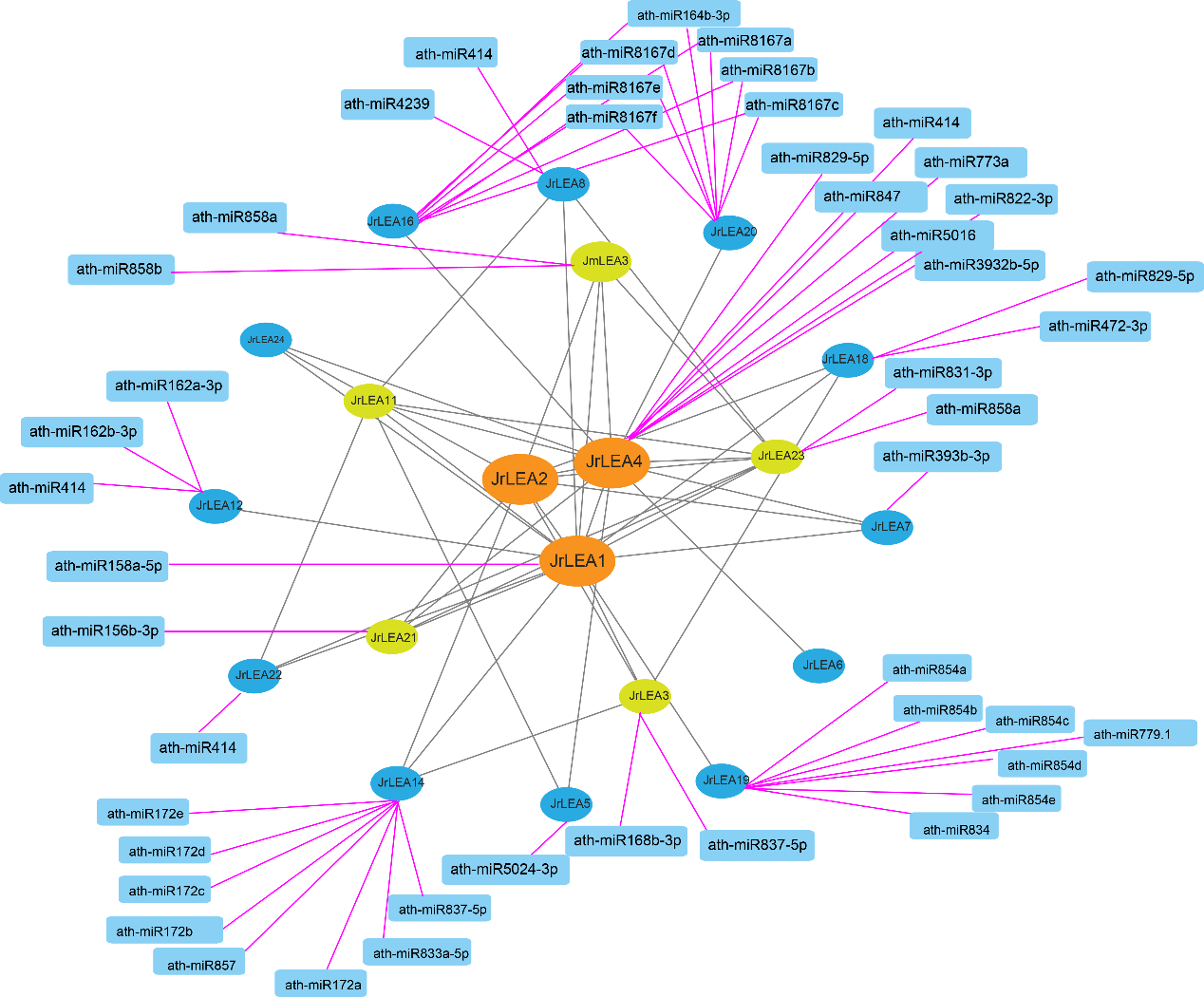
**

**Figure S2.** Expression patterns of *LEA* genes without collinearity in four selected organs of two *Juglans* species. The colored scale reflects gene expression levels.

**
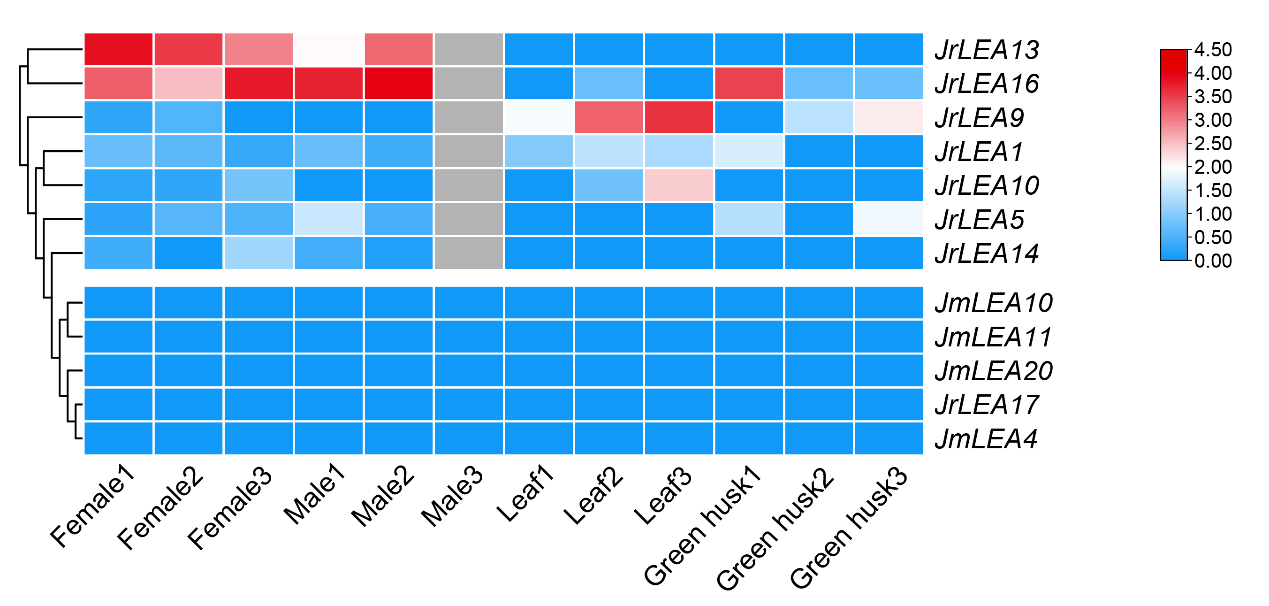
**

**Figure S3.** Expression patterns of *LEA* genes without collinearity under biotic stress of *J. regia*. F26 indicated anthracnose-resistant varieties, F423 indicated anthracnose-susceptible varieties. The colored scale reflects gene expression levels.


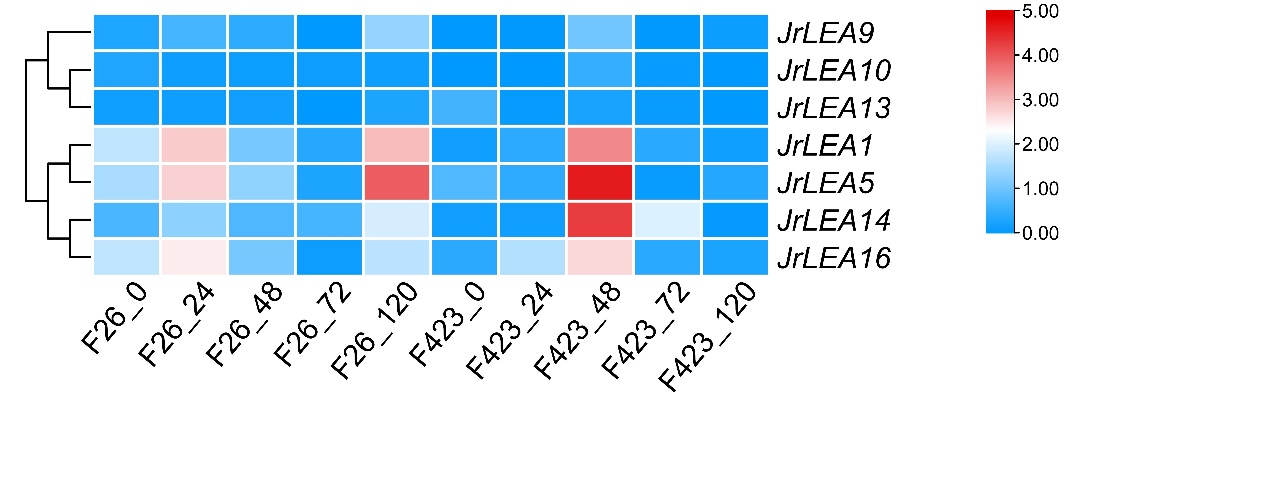

Supplement: Supplementary file 1 — Additional file 1: Figure S1. Protein interaction network and schematic representation of the regulatory network relationships between the putative miRNAs and their targeted LEA genes. Elliptics represent proteins, rectangles represent miRNAs. The black line indicates protein interactions and the pink line indicates miRNA targeting of LEA genes. Figure S2. Expression patterns of LEA genes without collinearity in four selected organs of two Juglans species. The colored scale reflects gene expression levels. Figure S3. Expression patterns of LEA genes without collinearity under biotic stress of J. regia. F26 indicated anthracnose-resistant varieties, F423 indicated anthracnose-susceptible varieties. The colored scale reflects gene expression levels. [file 12870_2023_4096_MOESM1_ESM.docx]
